# A Rapid Magnetic Solid Phase Extraction Method Followed by Liquid Chromatography-Tandem Mass Spectrometry Analysis for the Determination of Mycotoxins in Cereals

**DOI:** 10.3390/toxins9040147

**Published:** 2017-04-21

**Authors:** Giorgia La Barbera, Anna Laura Capriotti, Chiara Cavaliere, Patrizia Foglia, Carmela Maria Montone, Riccardo Zenezini Chiozzi, Aldo Laganà

**Affiliations:** Department of Chemistry, University of Rome “La Sapienza”, 00185 Roma, Italy; giorgia.labarbera@uniroma1.it (G.L.B.); annalaura.capriotti@uniroma1.it (A.L.C.); chiara.cavaliere@uniroma1.it (C.C.); patrizia.foglia@uniroma1.it (P.F.); carmelamaria.montone@uniroma1.it (C.M.M.); aldo.lagana@uniroma1.it (A.L.)

**Keywords:** mycotoxins, aflatoxins, ochratoxin A, zearalenone, magnetic solid phase extraction, graphitized carbon black, liquid chromatography-tandem mass spectrometry, cereals, wheat, maize

## Abstract

Mycotoxins can contaminate various food commodities, including cereals. Moreover, mycotoxins of different classes can co-contaminate food, increasing human health risk. Several analytical methods have been published in the literature dealing with mycotoxins determination in cereals. Nevertheless, in the present work, the aim was to propose an easy and effective system for the extraction of six of the main mycotoxins from corn meal and durum wheat flour, i.e., the main four aflatoxins, ochratoxin A, and the mycoestrogen zearalenone. The developed method exploited magnetic solid phase extraction (SPE), a technique that is attracting an increasing interest as an alternative to classical SPE. Therefore, the use of magnetic graphitized carbon black as a suitable extracting material was tested. The same magnetic material proved to be effective in the extraction of mycoestrogens from milk, but has never been applied to complex matrices as cereals. Ultra high–performance liquid chromatography tandem mass spectrometry was used for detection. Recoveries were >60% in both cereals, even if the matrix effects were not negligible. The limits of quantification of the method results were comparable to those obtained by other two magnetic SPE-based methods applied to cereals, which were limited to one or two mycotoxins, whereas in this work the investigated mycotoxins belonged to three different chemical classes.

## 1. Introduction

Mycotoxins are secondary metabolites produced by various filamentous fungi, mainly species of *Aspergillus*, *Fusarium*, and *Penicillium*, but also *Claviceps* and *Alternaria* [1]. These molds may grow under a wide range of climatic conditions on several agricultural commodities, including cereals, oleaginous seeds, spices, and coffee, both pre- and post-harvest (e.g., during storage) [2]. Some fungi produce a single mycotoxin, whereas others may produce many toxic compounds, which may be shared across fungal genera. 

There are about 400 known mycotoxins that exhibit a great structural diversity [2]. However, only a few of them are considered to be of agricultural importance [3]. Indeed, the main three genera of fungi, *Aspergillus*, *Fusarium*, and *Penicillium*, produce mycotoxins belonging to five relevant groups to the food industry: aflatoxins (AFs) produced by *Aspergillus* species, ochratoxin A (OTA) produced by both *Aspergillus* and *Penicillium* species, and fumonisins—specifically trichothecenes and resorcyclic lactones (zearalenones)—all produced mainly by *Fusarium* species [4,5,6].

Human exposure to mycotoxins occurs mostly through the intake of contaminated food and beverages, and to a minor extent through dermal contact and inhalation [1]. Mycotoxin occurrence in food is due to direct contamination of plant materials or products, or due to carry over of mycotoxins and their metabolites in foods (e.g., meat, milk, eggs) obtained from animals fed with contaminated feed [2].

The consumption of mycotoxin-contaminated food rarely determines acute toxicity [7]; nevertheless, a wide range of adverse effects for human and animal health, including carcinogenic, mutagenic, estrogenic, and immunosuppressive effects, has been demonstrated [8,9]. According to the International Agency for Research on Cancer system of classification, AFs are carcinogenic to humans (group 1) [5,10], whereas OTA is possibly carcinogenic to humans (group 2B), and zearalenone (ZEN) is not carcinogenic to humans (group 3) [5]. However, ZEN is well known for its estrogenic effect. Furthermore, OTA exposure has been related to nephropathies and other adverse health effects [11]. Finally, various mycotoxins might co-contaminate food, with possible detrimental additive and/or synergic effects on human and animal health [12].

In order to limit mycotoxin exposure and protect consumer and animal health from adverse effects, many countries have adopted regulations and maximum admissible levels (MLs) for the most prevalent and hazardous mycotoxins in certain commodities that are more prone to fungal proliferation [6,7]. Mycotoxins also have a negative impact on world trade: according to the annual report of the Rapid Alert System for Food and Feed [13], mycotoxins were the main hazard category for border rejection notifications in the European Union (EU) in 2015. Most of the notifications on mycotoxins in food were related to the presence of AFs (421/475 notifications), with a significant increase compared to 2014, whereas 42 notifications were due to OTA occurrence and the others (mainly to fusariotoxins).

Cereals, especially maize and bakery products, are one of the commercial categories frequently affected by mycotoxin presence [6]. The EU has fixed MLs for some mycotoxins in cereals and derived products, namely 2 and 4 µg kg^−1^ for AFB1 and the sum of the 4 AFs (AFB1, AFB2, AFG1, and AFG2), respectively, and 3 µg kg^−1^ for OTA. For ZEN, an ML of 75 µg kg^−1^ has been set for cereals and cereal flour, with the exception of maize, for which the ML is 100 µg kg^−1^ [14].

Methods for mycotoxin determination may roughly be classified as chromatographic-based, immunological-based, and sensor-based [3]. Chromatographic methods are generally used for confirmation purposes, whereas the other two method categories are often employed for screening analysis. Liquid chromatography, including ultra-high performance liquid chromatography (UHPLC), is generally preferred to gas chromatography for its versatility and is now considered the standard separation technique for mycotoxin analysis [3]. For detection, mass spectrometry (MS) and fluorescence (FD) are the gold standard against which all other methods are compared. In particular, tandem mass spectrometry (MS/MS) is the technique of choice for most authors [1].

Cereals are complex food matrices. Therefore, sample pre-treatment and/or clean-up and enrichment steps are generally required for most chromatographic methods. Although analysis time and cost are increased in this case, the analytical method benefits from increased sensitivity and robustness (e.g., reducing column blockage and contamination) [3]. After a preliminary solvent extraction from the solid matrix (generally with a mixture of acetonitrile or methanol and water), the mycotoxin extract is generally cleaned up/enriched by solid phase extraction (SPE). A wide variety of solid phases has been used, from the common C18-siclica bonded materials to the specific immunoadsorbent materials [3]. Recently, dispersive SPE, in a magnetic mode using nanoparticles, is attracting increasing scientific interest [15,16]. The mechanisms occurring in magnetic SPE (mSPE) are analogous to those observed in classical on-column SPE, where the interactions between target molecules and adsorbent functional groups determine the efficiency of the system. Certainly, the matrix composition also affects the selection of the best combination adsorbent—elution mixture [16]. In mSPE, the dispersion of the magnetic material into the solution containing the target molecules assures a continuous and dynamic contact with the adsorbent surface, leading to a more efficient analyte retention. The separation of the magnetic material with the adsorbed analytes from the solution is then realized by applying a magnet outside the vessel (e.g., on the bottom), avoiding centrifugation or filtration steps. Finally, after eventual washings, analytes are eluted from the magnetic material by a proper solvent mixture.

The mSPE technique followed by HPLC-FD analysis was already employed to extract mycotoxins from cereals. Magnetic nanoparticles coated by 3-(trimethoxysilyl)-1-propanethiol and different functionalizations were used to extract OTA from cereals [17] and AFB1 and AFB2 [18] from corn and rice samples.

In a previous study [19], the capability of magnetic graphitized carbon black (mGCB) in mycoestrogen extraction from milk samples was successfully tested. To this study’s authors’ best knowledge, that was the first application of mGCB in milk. In the present work, the same magnetic adsorbing material was employed to extract the main and most dangerous mycotoxins (namely AFB1, AFB2, AFG1, AFG2, OTA, and ZEN) from corn (*Zea mays*) meal and durum wheat (*Triticum durum*) flour. The extract was then analyzed by UHPLC-MS/MS with an electrospray (ESI) source. The method was suitably modified and validated in the new complex matrices. It was rapid and provided satisfying process efficiency (PE) and suitable limits of quantification (LOQs).

## 2. Results and Discussion

### 2.1. The Magnetic GCB Adsorbent Material

mGCB was chosen based on previous work [19], where its suitability for the extraction of mycoestrogens from milk was demonstrated. Moreover, in the past, GCB was also used in classical SPE mode to extract ZEN [20] and the four AFs [21] from maize.

Generally, most of the carbon-based materials used in aqueous environment need an oxidation step to improve their wettability and limit aggregation phenomena [22,23]. GCB is easily dispersed in water because of the presence of polar heterogeneities in its structure; nevertheless, a mild oxidation (1%, *w*/*w*) helped in reducing the elution solvent volume [19]. However, when treated in stronger oxidizing conditions, up to obtaining a 10% (*w*/*w*) oxidation, GCB lost most of its retention capability (data not shown).

The GCB characteristics before and after magnetization, as well as batch-to-batch preparation reproducibility, were previously assessed [19].

### 2.2. Samples

Most steps of the analytical method were developed using corn meal samples, and only successively validated for wheat flour samples. These two cereal flours contain nearly the same lipid amount, whereas protein and carbohydrate contents are significantly different (see Supplementary Table S1). Therefore, s-lensthe ESI matrix effects (MEs) on the analytes could also differ. Furthermore, the maize plant is very susceptible to contamination by *Fusarium* species [24], so it is generally more affected by certain mycotoxin contamination than wheat (in particular ZEN contamination) [6]. Indeed, during method development, the problem of finding blank samples emerged, since large amounts of ZEN were detected in most corn meal samples, thus leading to recovery (RE) and ME overestimate (up to 300%). Therefore, before matrix spiking, blank analysis of every new sample batch was performed to verify the absence of the investigated mycotoxins. Moreover, for each new sample set, a blank sample was randomly injected to verify the absence of possible carryover.

### 2.3. Sample Preparation

Generally, acetonitrile/water mixtures in the *v*/*v* ratios ranging from 75:25 up to 85:15 are used to extract mycotoxins from cereal samples; in some cases, acetic or formic acid up to 1% (*v*/*v*) is added to the mixture [1]. In the present work, before the mSPE procedure, corn meal and durum wheat flour samples were extracted with acetonitrile/water/formic acid 80:19.8:0.2 (*v*/*v*/*v*). In preliminary experiments, a neutral mixture and a mixture containing 1% formic acid were employed. However, high acid amounts had a detrimental effect on AF REs, whereas neutral mixture caused a slight decrease in the REs of OTA and ZEN (data not shown).

Different sample to mGCB ratios were tested, to obtain the highest overall PE values, i.e., taking into account both RE and ME. Keeping the magnetic material amount to 50 mg, experiments (three replicates for each condition) using 1000 mg, 500 mg, and 250 mg of maize meal were performed. In all three cases, the spiking level was 5 ng g^−1^ for the four AFs and OTA and 250 ng g^−1^ for ZEN. Results, which are reported in Supplementary Table S2, showed that the smallest sample amount allowed obtaining the best PE values for most mycotoxins. Moreover, with the lowest sample to adsorbent ratio, signal suppression for OTA was significantly reduced; the ME led to a moderate signal enhancement only for ZEN.

Five mL of dichloromethane/methanol 80:20 (*v*/*v*) containing 0.2% formic acid was used to elute the retained analytes from mGCB. A mixture dichloromethane/methanol, either neutral or containing up to 0.2% of a weak acid, was generally used to elute the analytes from GCB in classical SPE mode. In the analysis of maize samples, ZEN and its derivatives were eluted from GCB using a neutral dichloromethane/methanol 80:20 (*v*/*v*) mixture [25], while AFs were extracted with the same mixture containing acid [21]. In this case, the elution conditions were chosen to enhance the REs of the AFs, which are the natural least abundant and most toxic mycotoxins among the selected ones. Furthermore, the presence of acid in the elution mixture could also enhance the REs of compounds, such as OTA, establishing electrostatic interactions with the GCB surface [26].

At the end of the sample preparation procedure, the solvents were removed by evaporation from the eluate, and the residue was reconstituted with 250 µL of methanol/water 80:20 (*v*/*v*) containing 5 mmol L^−1^ ammonium formate. The choice of the reconstitution mixture appeared to be a critical point. Initially, a mixture of acetonitrile/water 50:50 (*v*/*v*) containing ammonium formate was used. However, REs were low for most of the analytes (see Table 1), very likely due to solubility issues. A larger amount of acetonitrile did not significantly improve such results, since the little RE increase was associated with worse MEs. Finally, the replacement of acetonitrile with methanol gave the best results, also in terms of chromatographic peak broadening.

### 2.4. Liquid Chromatography-Tandem Mass Spectrometry Analysis

**Figure 1:**
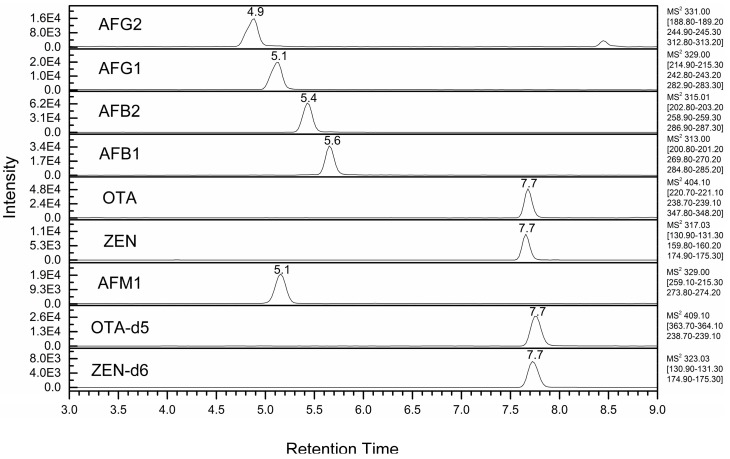
Extracted ion chromatograms (sum of three transition pairs for each analyte) of a corn meal sample spiked with the analytes at 0.5 × ML.

Analytes were separated onto a reversed-phase C18 chromatographic column. In the first experiments, a Thermo Fisher Hypersil Gold C18 column (50 × 2.1 mm i.d., 1.9 μm particle size) was used, and different elution gradients were tested to reduce signal suppression due to MEs. However, in corn samples, OTA ME was ca. 50% in all the chromatographic conditions (data not shown), even reducing the gradient rate or eluting the analyte in isocratic conditions. Such large ME was attributed to the effect of some coextracted lipids eluting at retention times very close to that of OTA. Indeed, in previous works on mycotoxin determination in maize meal [25,27], the extract was filtered through a C18 cartridge before GCB SPE clean-up to retain phospholipids and most triglycerides. In the present work, lower MEs were obtained only by using a longer column, namely a 100 mm Cortecs UPLC C18+ column by Waters (see Supplementary Table S3). Figure 1 shows the extracted ion chromatograms relative to the six investigated analytes in a corn meal extract, whereas Figure S1 shows the extracted ion chromatograms relative to the six investigated analytes in a wheat flour extract.

Analyte-specific acquisition parameters (e.g., S-lens, precursor ions, MRM transitions and collision energy) were optimized by directly infusing 1 ng μL^−1^ individual mycotoxin standard solution prepared in water/methanol 50:50 (*v*/*v*) at 10 µL min^−1^ (see Table 2). General ESI source tune parameters (i.e., spray voltage, gas pressures, and source temperatures) were optimized by simultaneously introducing, through a tee-junction, the same 1 ng μL^−1^ individual mycotoxin standard solution at 10 µL min^−1^ and water/methanol 50:50 (*v*/*v*) containing 5 mmol L^−1^ ammonium formate and 0.1% formic acid at 300 µL min^−1^ flow-rate. The chosen parameters were a compromise between the optimal ones determined for each analyte.

For each analyte, the unambiguous identification was based on comparison with the authentic standard (retention times, relative intensity ratios of MRM transition pairs) following the criteria reported in the Decision 2002/657/EC [28].

### 2.5. Reuse of MGCB

As in a previous work on magnetic nanoparticles coated with polydopamine [29], the reuse of mGCB was evaluated, but it was not convenient due to the large amount of solvent required for mGCB effective washing and the loss of material during such operation.

### 2.6. Method Performance

The MLs fixed by EU for AFs in cereals are 2 µg kg^−1^ for the most dangerous and widespread AFB1 and 4 µg kg^−1^ for the sum of AFB1, AFB2, AFG1 and AFG2. For method development, the worst scenario was prospected, i.e., each of the four AFs at 1 µg kg^−1^ concentration.

For laboratory method validation, RE and ME were determined at three spiking levels in both corn meal and durum wheat flour samples, according to Equations (1) and (2) (see Table 3). The product of RE and ME provides the overall PE (Equation (3)). The RE relative standard deviations were below 17% for all the analytes.

Recoveries were >67% at the lowest fortification level. The MEs of signal suppression affected in particular AFB1 and OTA. The only signal enhancement ME was observed for ZEN, however it cannot be excluded that this signal enhancement was due to a natural contamination below method detection limit (MLOD).

The trueness of the method was assessed by means of apparent REs (Equation (4)) at three different fortification levels, whereas intra-day and inter-day laboratory precision were determined by performing recovery experiments (*n* = 6) at 0.5 × ML in the same day and in six consecutive days, respectively (see Table 4). Apparent REs were higher for the two mycotoxins whose deuterated ISs were available, i.e., OTA and ZEN, whereas for the four AFs, the hydroxylated Phase I AFB1 metabolite, namely AFM1, was used as IS.

The equations and coefficient of determination (R^2^) obtained for the standard and the two matrix-matched calibration graphs are reported in Supplementary Table S4.

The MLODs and MLOQs were determined as described in the Experimental section (see Table 5), since by operating in MRM mode with the last generation triple quadrupole mass spectrometers, it is quite common to obtain MRM signals without noise [8]. The mSPE technique was used to extract AFB1, AFB2 [18] and OTA [17] from cereals. Compared to the work by Hashemi et al. [18], which obtained MLOQs around 0.2 and 0.05 µg kg^−1^ for AFB1 and AFB2, respectively, the MLOQs obtained in the present work for AFB2 are slightly higher. Compared to the work by Mashhadizadeh et al. [17], the limits for OTA are three times higher. However, it should be considered that both these works used HPLC-FD for determination, thus the criteria for MLOD and MLOQ estimation are different, and they analyzed from one up to two mycotoxins.

Moreno et al. [30] used magnetic nanoparticles coated with a layer of octadecyl group-modified silica containing multiwalled carbon nanotubes to extract ZEN and its derivative from maize, followed by LC-MS analysis. They used only 5 mg of magnetic nanoparticles; however, a comparison with this method is difficult, since it is not clear if 6 g or 10 g sample aliquot was used in the sample preparation procedure. Moreover, LODs and LOQs are provided in µg L^−1^, thus the concentration is not clearly referred to maize amount. Ethylene glycol bis-mercaptoacetate modified silica coated magnetic nanoparticles were also used to extract AFs from wheat before spectrofluorometric analysis [31]; the MLODs (0.07 µg kg^−1^) were comparable to those obtained in the present work, whereas the MLOQs (0.24 µg kg^−1^) were higher.

### 2.7. Sample Analysis

One of the problems arising during method development was to find samples free of ZEN, which, indeed, contaminates most of the corn meal samples [24,25]. OTA was also detected in some samples, though at concentrations below MLOQ. In a short survey carried out on 10 corn meal samples, OTA was detected in one sample at 1.3 µg kg^−1^ level, whereas it was detected at values >MLOD but <MLOQ in the other two samples. In eight out of 10 samples, ZEN was detected at values >MLOD but <MLOQ, and in one sample at 72.9 µg kg^−1^ level. Quantification was made by matrix-matched calibration (results are shown in Table S5). None of the investigated mycotoxins were detected in the five durum wheat flour samples.

## 3. Conclusions 

In this work, the suitability of mGCB for the extraction of AFB1, AFB2, AFG1, AFG2, OTA, and ZEN from corn meal and durum wheat flour samples was demonstrated. The overall process efficiency of the developed method was a compromise between performance and easiness and rapidity of application. Compared with a classic on-column SPE, the time required for a single extraction was about the same. Nevertheless, mSPE was less labor intensive, and more than ten extractions can be managed simultaneously.

Even if in the present work, the limits of quantification were comparable to or higher than those of other mSPE methods. However, this method allows for the simultaneous investigation of a larger number of mycotoxins. Moreover, due to the different detection technique (fluorescence in the other works and tandem mass spectrometry in the present one), MLOQ calculation modes are very different.

This is the first application of mGCB in an mSPE procedure for extraction of mycotoxins from cereals. The potentiality of this material has been exploited before for the extraction of mycotoxins belonging to the same chemical class, but from milk.

## 4. Materials and Methods 

### 4.1. Chemicals and Reagents

Organic solvents of analytical grade, formic acid, nitric acid, ammonium formate, hydrochloric acid (reagent grade), iron (III) chloride hexahydrate, ethylene glycol, trisodium citrate, sodium acetate, poly(ethyleneglycol)-10k, and GCB (Supelclean ENVI-Carb, surface area: 100 m^2^/g, particle size: 120/400 mesh) were obtained from Sigma-Aldrich (St. Louis, MO, USA). LC-MS grade methanol and ultrapure water (resistivity 18.2 MΩ cm) were obtained from Sigma Aldrich and used for LC mobile phase.

Pure (purity ≥98%, unless differently specified) standards of the analytes AFB1, AFB2, AFG1, AFG2, OTA, and ZEN (purity ≥99%) were purchased from Sigma-Aldrich. The standards of aflatoxin M1 (AFM1) and deuterated OTA (OTA-d5) acquired from Sigma-Aldrich, and deuterated ZEN (ZEN-d6) acquired from Wellington Laboratories (Toronto, ON, Canada) were used as internal standards (ISs).

Individual stock standard solutions of the analytes were prepared at 200 ng µL^−1^ in methanol. A composite working standard solution of the six analytes was prepared in methanol at 10 pg µL^−1^ for AFB1, AFB2, AFG1, and AFG2, 30 pg µL^−1^ for OTA, and 750 pg µL^−1^ for ZEN. This mixture was renewed every two weeks. All the solutions were stored in the dark at −20 °C and brought to room temperature before use.

#### Safety Considerations

AFs and OTA are carcinogenic and possibly carcinogenic compounds to humans, respectively. Therefore, handling standard solutions and extracts requires extreme care. Gloves and other protective clothing were worn as a safety precaution during the handling of the mycotoxins. Solid AF standards were handled in a glove box. Glassware used for standards or samples was soaked in 3% aqueous sodium hypochlorite to destroy mycotoxin residue before cleaning and re-use. When possible, disposable plastic material was used. To avoid degradation, mycotoxins were protected from daylight during sample preparation and the standard solutions were kept in amber vials.

### 4.2. Magnetic Graphitized Carbon Black Preparation

As in a previous work [19], mGCB was prepared by adapting a literature protocol for carbon nanotubes magnetization [32]. Briefly, 0.40 g of GCB was first added to 50 mL of concentrated nitric acid under agitation for 7 h at room temperature. After that, the material was washed with distilled water until the discarded water reached neutral pH, and then dried overnight at 50 °C. For magnetization, 0.30 g of GCB was dispersed into 80 mL of ethylene glycol and added with 1.62 g of iron (III) chloride hexahydrate, 0.30 g of trisodium citrate, 7.20 g of sodium acetate, and 2.00 g of poly(ethylene glycol). After 3 h sonication (with a 37 kHz Elmasonic S 60 H by Elma, Singen, Germany), the obtained mixture was sealed in an autoclave for 10 h at 200 °C. The resulting mGCB microparticles were allowed to cool at room temperature for 3 h, then washed with 30 mL ethanol followed by 30 mL distilled water six times. Finally, the mGCB was dried in an oven at 80 °C for 3 h; after cooling, it was stored in a glass flask at room temperature until use.

### 4.3. Characterization of Graphitized Carbon Black Material

Transmission electron microscopy, Fourier transform infrared spectroscopy spectra, thermogravimetric analysis, porosimetry, and specific surface area analysis were used to characterize the GCB material at each preparation step, i.e., before and after treatment with nitric acid, and after magnetization [19].

### 4.4. Samples and Extraction Protocol

Samples of *Triticum durum* flour and *Zea mays* meal were obtained from local markets of the Lazio region (Italy).

Sample aliquot of 250 mg was placed in a 15 mL-polypropylene centrifuge tube and added with 2.5 mL of the extracting mixture constituted by acetonitrile/water/formic acid 80:19.8:0.2 (*v*/*v*/*v*). The tube was vortexed (with a Digital Vortex-Genie 2 by Scientific Industries, Bohemia, NY, USA) at 2000 rpm for 3 min, then placed in an ultrasonic bath for 10 min, and finally centrifuged at 12,500× *g* for 15 min at 4 °C. After that, the supernatant was transferred to a 50 mL-polypropylene centrifuge tube containing 50 mg of mGCB previously conditioned. The conditioning procedure consisted in adding to the weighted material 5 mL of methanol followed by 5 mL of ultrapure water. Each time, after vortexing for 30 s, the material was allowed to settle down by magnetic decantation (using a permanent magnetic disk Nd-Fe-B, 25 mm × 5 mm, by Supermagnete, Gottmadingen, Germany), and the solvent was removed.

The mixture extract-mGCB was diluted with 22.5 mL ultrapure water and vortexed at 900 rpm for 30 min to promote the analyte adsorption onto the magnetic microparticles. At this point, as well as during the following protocol steps, the solvent was removed by magnetic decantation with a permanent magnetic disk placed on the bottom of the tube; the mGCB was washed with 4 mL of ultrapure water by vortexing for 30 s at 2800 rpm. Water was removed and 250 µL of methanol was added to eliminate residual water. After 30 s-manual shaking, methanol was removed. Mycotoxins were eluted from mGCB by 5 mL of dichloromethane/methanol 80:20 (*v*/*v*) containing 0.2% formic acid under vortexing for 3 min at 2000 rpm. The supernatant was collected in a clean 20 mL round bottom glass vial and the solvent was removed by a gentle nitrogen stream in a water bath at 37 °C. When required, the three ISs were added to the extract before solvent removal. Finally, the residue was reconstituted with 250 µL of methanol/water 80:20 (*v*/*v*) containing 5 mmol L^−1^ ammonium formate; after 30 s vortexing, the solution was placed in an ultrasonic bath for 10 s (3 times). Before transferring the extract in an autosampler vial, it was centrifuged (by a MicroCL 21R centrifuge, Thermo Scientific, Waltham, MA, USA) at 21,000× *g* for 5 min to eventually sediment suspended particles and/or remove residual fats.

To artificially fortify mycotoxin-free samples before extraction (e.g., for preparation of the matrix-matched calibration solutions and RE experiments), and promote analyte dispersion onto the whole sample, the procedure was as follows. Two hundred and fifty mg of durum wheat flour or corn meal was soaked in 250 µL of acetone containing the required amount of the analyte working standard mixture. Then, the sample was placed for 1 h in a ventilated oven at 40 °C to let the organic solvent evaporate. Finally, the spiked sample was extracted as reported above. The fortification levels were at ML, i.e., 1 µg kg^−1^ for each AF, 3 µg kg^−1^ for OTA and 750 µg kg^−1^ for ZEN (obtained by adding 25 µL of the working solution, 10 pg µL^−1^ each AF, 30 pg µL^−1^ OTA, and 750 pg µL^−1^ ZEN), and its multiples or fractions.

### 4.5. Liquid Chromatography-Tandem Mass Spectrometry Conditions

The UHPLC/ESI-MS/MS system was from Thermo Fisher Scientific (Bremen, Germany) and was constituted by a triple quadrupole mass spectrometer, mod. TSQ (triple stage quadrupole) Vantage EMR^TM^ (enhanced mass range), coupled to an UHPLC system Ultimate 3000 binary pump via a heated ESI source. The software Xcalibur^TM^ 2.2 (Thermo Fisher Scientific, Bremen, Germany) was used for LC-MS data acquisition and processing.

Sample aliquots of 10 µL were injected via the UHPLC autosampler. The six mycotoxins were separated onto a reversed-phase Cortecs UPLC C18+ column (100 mm × 2.1 mm i.d., 1.6 μm particle size), preceded by a VanGuard pre-column (5 mm × 2.1 mm i.d.) packed with the same stationary phase (Waters Milford, MA, USA). The column was thermostatted at 40 °C. The mobile phase was water (A) and methanol (B), both containing 5 mmol L^−1^ ammonium formate and 0.1% formic acid; the flow-rate was 300 µL min^−1^. The elution gradient was the following: after 0.5 min at 15%, B was linearly increased to 35% in 1 min, then to 68% in 3.5 min, and finally to 75% in 3 min. To rinse the column, B was brought to 98% in 1 min, and held constant for 3 min. After bringing B back to 15% in 0.5 min, the column was allowed to equilibrate for 5.5 min.

MS Data were acquired in multiple reaction monitoring (MRM) mode, by operating ESI source in both positive (for the four AFs, OTA, and the corresponding ISs) and negative (for ZEN and its IS) ionization mode. The tune parameters were set as follows: spray voltage, +3.2/−2.8 kV; vaporizer temperature, 280 °C; capillary temperature, 220 °C; sheath gas pressure, 50 (arbitrary units, a.u.); sweep ion gas pressure (+) 0/(−) 1 (a.u.); auxiliary gas pressure, 25 (a.u.). For each compound, from two to three MRM transitions were monitored (see Table 1).

Monthly, the calibration solutions provided by Thermo Fisher Scientific (range *m*/*z* 69-2800) were injected in infusion mode for mass calibration and resolution adjustments of the resolving lens and quadrupole.

### 4.6. Analytical Method Performance

To assess the performance of the developed analytical method, overall PE, trueness and precision (both intra-day and inter-day precision), MLODs, and MLOQs were considered.

#### 4.6.1. Process Efficiency (Recovery and Matrix Effect)

RE, ME, and PE were evaluated as in a previous work [29]. Blank matrix solutions spiked with mycotoxin standards before and after extraction were named as sample set 1 and set 2, respectively, whereas neat standard solutions prepared in pure solvents were named as sample set 3. For each analyte, the absolute peak area was measured. The RE was assessed according to the following equation:

RE (%) = (Area set1/Area set2) × 100(1)

ME was estimated according to the following equation:

ME (%) = (Area set2/Area set3) × 100(2)

PE, which is the product between RE and ME, was estimated according to the following equation:

PE (%) = (Area set1/Area set3) × 100(3)

The fortification levels used to assess PE were three (*n* = 6 for each level). For matrix solutions (sample sets 1 and 2), the levels were: ML, 0.5 × ML and 2 × ML. For standards solutions (sample set 3), these levels corresponded to 1 pg µL^−1^ of the four AFs, 3 pg µL^−1^ of OTA, and 75 pg µL^−1^ of ZEN, defined as standard ML (sML), 0.5 × sML and 2 × sML.

#### 4.6.2. Calibration Graphs

For each mycotoxin, both six-point standard and matrix-matched calibration graphs were constructed. Standard solutions were prepared in water/methanol (50:50, *v*/*v*) at 0.2 × sML, 0.3 × sML, 0.5 × sML, sML, 2 × sML, and 4 × sML concentration levels. Matrix-matched solutions were prepared by spiking analyte-free samples before extraction at 0.2 × ML, 0.3 × ML, 0.5 × ML, ML, 2 × ML, and 4 × ML levels. The same amount of the three ISs was added to all the solutions, i.e., 2 pg µL^−1^ for AFM1, 6 pg µL^−1^ for OTA-d5 and 150 pg µL^−1^ for ZEN-d6.

Each solution was prepared in duplicate and injected twice, starting from the lowest up to the highest concentration level; finally, the results were averaged to give rise to a single calibration graph for each mycotoxin.

For each analyte, the combined ion current profile for the selected transitions was extracted from the LC-MRM dataset; the resulting traces were smoothed (Gaussian type, 7 points) by applying the automatic processing smoothing of XcaliburTM software.

The analyte to the corresponding IS peak area ratio versus the analyte concentration was plotted, considering the sum of all the MRM transitions to measure the areas. Unweighted regression lines for standard and matrix-matched calibration graphs were calculated using XcaliburTM QuanBrowser (Thermo Fisher Scientific).

#### 4.6.3. Trueness and Precision 

To assess the trueness of the developed method, each mycotoxin apparent recovery was calculated according to the following equation:

Apparent RE (%) = [(Area analyte set 1/Area IS set 1)/(Area analyte set 2/Area IS set 2)] × 100(4)

i.e., by comparing the analyte to IS peak area ratios in free-analyte flour samples spiked before and after the extraction procedure. The samples were spiked at the same levels used for PE assessment; the amounts of the three ISs was the same used in the calibration experiments. For each spiking level, six replicates were performed.

Intra-day (repeatability) and inter-day (reproducibility) were used to evaluate within laboratory precision of the developed analytical method. The relative standard deviation of the apparent recovery values of six spiked samples, at 0.5 × ML concentration, analyzed in the same day (RSDr) and in six consecutive days (RSD_R_) were used to estimate the intra-day and inter-day laboratory precision, respectively.

#### 4.6.4. Method Limits of Detection and Quantification

MLODs and MLOQs of the analytes were assessed as reported in previous works [8,33]. Briefly, a first estimation was done in the classical way, i.e., according to the following equations:

MLOD = 3 × σ/*S* (5)

and

MLOQ = 10 × σ/*S* (6)

where σ is the standard deviation of the intercept and *S* the slope of the matrix-matched calibration graph. For MLOQ calculation, the peak area obtained by the sum of all the MRM transitions was used, whereas for MLOD calculation, the peak area obtained only by the second most intense MRM transition was considered.

After those calculations, the MLOD and MLOQ values obtained according to Equations (5) and (6), respectively, were verified. Therefore, corn meal and wheat flour samples were spiked with the six mycotoxins at levels very close to the extrapolated MLOQ values, and subject to the whole analytical procedure. For limits confirmation, the following equations were used:

MLOD = 3 × *S*/*N* (7)

and

MLOQ = 10 × *S*/*N* (8)

where *S/N* is the signal to noise ratio manually estimated by the LC-MRM data set, since the *S*/*N* provided by Thermo Xcalibur Qual Browser software by both INCOS noise method and manual noise region selection, was unlikely high.

## Figures and Tables

**Table 1:** Recovery (RE, %) and matrix effect (ME, %) obtained from the extraction of corn meal samples spiked with 0.5 µg kg^−1^ of each AF, 1.5 µg kg^−1^ of ochratoxin A (OTA) and 375 µg kg^−1^ of zearalenone (ZEN). The residue was reconstituted with three different mixtures containing 5 mmol L^−1^ ammonium formate: (I) acetonitrile/water 50:50 (*v*/*v*); (II) acetonitrile/water 80:20 (*v*/*v* II); methanol/water 80:20 (*v*/*v*).

Analyte	ACN/H_2_O 50:50		ACN/H_2_O 80:20		MeOH/H_2_O 80:20	
	RE	ME	RE	ME	RE	ME
AFG2	44	78	55	68	61	72
AFG1	53	74	50	73	61	70
AFB2	49	77	62	66	53	76
AFB1	45	73	67	56	64	65
OTA	61	63	64	68	56	66
ZEN	83	86	78	82	81	89

**Table 2:** Mycotoxin detection parameters.

Mycotoxin	Retention Time (Min)	Precursor Ion (*m/z*)	Product Ion ^1^ (*m/z*) (CE ^2^, eV)	S-Lens (V)
Positive polarity		[M + H]^+^		
AFG2	4.9	331	189 (42), 245 (31), **313** (25)	145
*AFM1* (IS)	5.1	329	259 (34), **273** (25)	145
AFG1	5.4	329	215 (33), **243** (27), 283 (24)	145
AFB2	5.6	315	203 (36), 259 (33), **287** (24)	155
AFB1	4.6	313	201 (30), 270 (26), **285** (23)	150
*OTA-d5* (IS)	7.7	409	363 (25), **239** (27)	
OTA	7.7	404	221 (36), **239** (26), 348 (13)	110
Negative polarity		[M − H]^−^		
*ZEN-d6* (IS)	7.7	323	131 (33), **175** (25)	140
ZEN	7.7	317	131 (33), 160 (34), **175** (25)	150

**^1^** In bold, the most intense transition; **^2^** CE, collision energy.

**Table 3:** Recovery (RE, %, *n* = 6) and matrix effect (ME, %) for AFs, OTA and ZEN in corn meal and durum wheat flour samples. Fortification levels were maximum limit (ML, i.e., 1 µg kg^−1^ for each AF, 3 µg kg^−1^ for OTA and 750 µg kg^−1^ for ZEN), 0.5 × ML and 2 × ML.

Analyte	0.5 × ML				ML				2 × ML			
	Corn		Durum Wheat		Corn		Durum Wheat		Corn		Durum Wheat	
	RE	ME	RE	ME	RE	ME	RE	ME	RE	ME	RE	ME
AFG2	78	76	74	86	67	79	69	79	71	84	67	88
AFG1	71	75	74	79	66	73	74	75	68	78	73	84
AFB2	69	76	68	78	63	85	76	86	74	89	71	76
AFB1	73	68	71	70	74	69	73	68	72	69	69	73
OTA	67	72	73	67	83	68	76	71	79	69	81	68
ZEN	78	87	79	102	89	94	82	116	84	104	88	108

**Table 4:** Trueness, intra-day and inter-day laboratory precision obtained by analyzing corn meal and durum wheat flour samples spiked with aflatoxins (AFs), OTA, and ZEN at maximum limit (ML, i.e., 1 µg kg^−1^ for each AF, 3 µg kg^−1^ for OTA and 750 µg kg^−1^ for ZEN), 0.5 × ML and 2 × ML. Results are averaged from *n* = 6, performed in the same day and in six consecutive days.

Analyte	Trueness						Precision (0.5 × ML)			
	0.5 × ML		ML		2 × ML		Intra-Day		Inter-Day	
	Corn	Wheat	Corn	Wheat	Corn	Wheat	Corn	Wheat	Corn	Wheat
AFG2	94	98	95	96	92	91	9	10	17	14
AFG1	93	99	97	95	98	96	8	12	7	9
AFB2	89	91	94	89	103	99	6	3	10	8
AFB1	90	93	94	90	106	101	10	7	8	10
OTA	99	97	98	100	96	97	7	4	11	9
ZEN	103	99	101	98	104	106	11	3	16	20

**Table 5:** Method limits of detection (MLODs) and quantification (MLOQs) estimated (est.) according to Equations (5) and (6) and confirmed (conf.) according to Equations (7) and (8).

Analyte	MLODs (µg kg^−1^)				MLOQs (µg kg^−1^)			
	Corn		Durum Wheat		Corn		Durum Wheat	
	Est.	Conf.	Est.	Conf.	Est.	Conf.	Est.	Conf.
AFG2	0.11	0.05	0.12	0. 05	0.38	0.20	0.43	0.15
AFG1	0.08	0.10	0.13	0.05	0.27	0.20	0.23	0.15
AFB2	0.09	0.05	0.14	0.05	0.29	0.10	0.43	0.10
AFB1	0.11	0.10	0.23	0.10	0.36	0.10	0.23	0.10
OTA	0.48	0.10	0.25	0.20	1.60	0.30	0.79	0.30
ZEN	10.2	1.0	4.2	2.2	33.8	1.0	34.6	2.2

## Supplementary Materials

The following are available online at www.mdpi.com/2072-6651/9/4/147/s1, Table S1: *Zea mays* meal and *Triticum durum* flour composition, Table S2: Recovery, Matrix Effect, and Process Efficiency using different corn meal amounts, Table S3: Matrix effects using two different C18 chromatographic columns, Table S4: Equations and coefficient of determination relative to standard and matrix-matched calibration curves, Table S5: Results of 10 corn meal sample survey, Figure S1: Extracted ion chromatograms (sum of three transition pairs for each analyte) of a wheat flour sample spiked with the analytes at 0.5 × ML.
