# Supplementary material for: A Rapid Magnetic Solid Phase Extraction Method Followed by Liquid Chromatography-Tandem Mass Spectrometry Analysis for the Determination of Mycotoxins in Cereals
Source: Toxins (Basel). 2017 Apr 21;9(4):147. doi: 10.3390/toxins9040147 (PMC5408221; doi:10.3390/toxins9040147)
Supplement: Supplementary file 1 [file toxins-09-00147-s001.pdf]

Supplementary Materials: A Rapid Magnetic Solid Phase Extraction Method Followed By Liquid Chromatography-Tandem Mass Spectrometry Analysis for the Determination of Mycotoxins in Cereals

Giorgia La Barbera, Anna Laura Capriotti, Chiara Cavaliere, Patrizia Foglia, Carmela Maria Montone, Riccardo Zenezini Chiozzi and Aldo Laganà

Table S1. *Zea mays* meal and *Triticum durum* flour composition according to Italian Research Center for Food and Nutrition (http://nut.entecra.it/646/tabelle_di_composizione_degli_alimenti.html).

Chemical composition (g/100 g)	*Zea mays*	*Triticum durum*
Water	12.5	12.5
Proteins	8.7	12.9
Lipids	2.7	2.8
Available carbohydrates	80.8	63.2
Starch	72.1	54.5
Soluble sugars	1.5	3.2

Table S2. Recovery (RE, %), Matrix Effect (ME, %) and Process Efficiency (PE, %, i.e. RE×ME) obtained using 50 mg of magnetic GCB and different corn meal amounts. See equations 1-3 in the manuscript for their calculation. Samples were spiked with 5 ng g^{-1} of the four AFs and OTA and 250 ng g^{-1} of ZEN, then extracted as described in the Experimental section. Results are an average of three experimental replicates.

Analyte	1000 mg corn meal			500 mg corn meal			250 mg corn meal		
	RE	ME	PE	RE	ME	PE	RE	ME	PE
AFG2	45	87	39	66	79	52	62	91	56
AFG1	52	82	43	69	77	53	74	72	53
AFB2	59	82	48	74	79	58	73	81	59
AFB1	56	92	52	71	86	61	73	71	52
OTA	79	29	23	79	28	22	87	70	61
ZEN	64	105	67	75	98	74	76	117	89

Table S3. Matrix Effect (ME, %) obtained by analyzing 0.25 g maize flour sample spiked after extraction at ML, i.e., 1 μg kg^{-1} for each AF, 3 μg kg^{-1} for OTA and 750 μg kg^{-1} for ZEN, and a standard solution containing the same nominal mycotoxin concentration. The same solution were analyzed using two different C18 chromatographic columns: I) a Hypersil Gold C18 column (50 × 2.1 mm i.d., 1.9 μm particle size); II) and a Cortecs UPLC C18+ column (100 mm × 2.1 mm i.d., 1.6 μm particle size). Results are an average of two technical replicates.

Analyte	Column I ME	Column II ME
AFG2	78	93
AFG1	72	84
AFB2	75	72
AFB1	76	77
OTA	52	72
ZEN	117	118

Table S4. Equations and coefficient of determination (R^2) relative to standard and matrix-matched calibration curves. Matrix-matched calibration solutions were prepared by spiking either corn meal and durum wheat flour samples before extraction and following the experimental procedure. The three ISs were also added in constant amount in all the solutions.

**Analyte**	**Standard calibration (R^2)**	**Corn meal matrix-matched calibration (R^2)**	**Durum wheat flour matrix-matched calibration (R^2)**
AFG2	y=63309x+1575.2 (0.9999)	y=31525x+2595.3 (0.9988)	y=52928x-3695 (0.9979)
AFG1	y=107106x+190.76 (0.9998)	y=47273x+1714.5 (0.9981)	y=64419x-5372 (0.9994)
AFB2	y=217290x+403.79 (0.9997)	y=137769x-771.69 (0.9998)	y=199913x-18956 (0.9979)
AFB1	y=151127x+396.28 (0.9996)	y=79617x+2426.9 (0.9989)	y=107350x-9890.8 (0.9994)
OTA	y=25883x-88597 (0.9911)	y=36269x-1348.5 (0.9998)	y=46954x-11043 (0.9992)
ZEN	y=285.45x-507.28 (0.9991)	y=0.0057x-127.18 (0.9968)	y=88.94x-917.2 (0.9976)

Table S5. Results on ten corn meal sample survey.

	**Samples (μg kg^{-1})**									
	**C1**	**C2**	**C3**	**C4**	**C5**	**C6**	**C7**	**C8**	**C9**	**C10**
AFG2	<MLOD	<MLOD	<MLOD	<MLOD	<MLOD	<MLOD	<MLOD	<MLOD	<MLOD	<MLOD
AFG1	<MLOD	<MLOD	<MLOD	<MLOD	<MLOD	<MLOD	<MLOD	<MLOD	<MLOD	<MLOD
AFB2	<MLOD	<MLOD	<MLOD	<MLOD	<MLOD	<MLOD	<MLOD	<MLOD	<MLOD	<MLOD
AFB1	<MLOD	<MLOD	<MLOD	<MLOD	<MLOD	<MLOD	<MLOD	<MLOD	<MLOD	<MLOD
OTA	<MLOD	<MLOD	< MLOQ	1.3	<MLOD	<MLOD	< MLOQ	<MLOD	<MLOD	<MLOD
ZEN	< MLOQ	< MLOQ	72.9	< MLOQ	< MLOQ	<MLOD	< MLOQ	< MLOQ	< MLOQ	< MLOQ

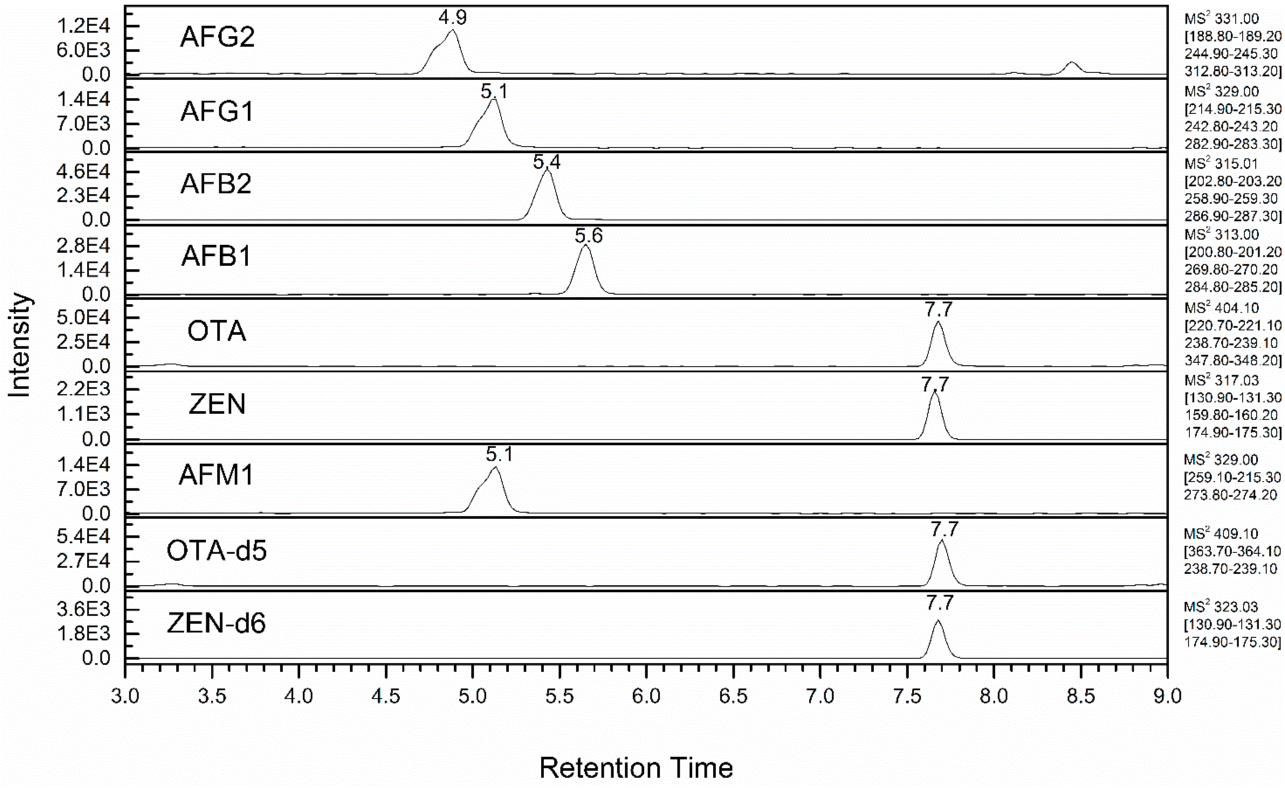

Figure S1. Extracted ion chromatograms (sum of three transition pairs for each analyte) of a wheat flour sample spiked with the analytes at 0.5×ML.
